# Intratumoral and peritumoral radiomics for preoperative prediction of pathological complete response to neoadjuvant immunochemotherapy in patients with esophageal squamous cell carcinoma

**DOI:** 10.1016/j.ejro.2026.100774

**Published:** 2026-06-17

**Authors:** Bo Zhao, Yaqi Wang, Haitao Zhu, Xinrun Cui, Xiaoting Li, Shaolei Li, Nan Wu, Yingshi Sun

**Affiliations:** a Key Laboratory of Carcinogenesis and Translational Research (Ministry of Education/Beijing), Department of Radiology, Peking University Cancer Hospital & Institute, Beijing 100142, China; b Key Laboratory of Carcinogenesis and Translational Research (Ministry of Education/Beijing), Department of Thoracic Surgery II, Peking University Cancer Hospital & Institute, Beijing 100142, China; c State Key Laboratory of Molecular Oncology, Department of Thoracic Surgery II, Peking University Cancer Hospital & Institute, Beijing 100142, China; d Department of Thoracic Surgery, Peking University Cancer Hospital Inner Mongolia Hospital, Cancer Hospital Affiliated to Inner Mongolia Medical University, Hohhot 010110, China

**Keywords:** Esophageal squamous cell carcinoma, Neoadjuvant immunochemotherapy, Pathological complete response, Radiomics, Peritumoral region

## Abstract

**Background:**

Pathological complete response (pCR) after neoadjuvant immunochemotherapy (nICT) confers a significant survival advantage in locally advanced esophageal squamous cell carcinoma (ESCC), yet remains difficult to anticipate preoperatively. This study aimed to develop and validate a noninvasive, multimodal model integrating intratumoral and peritumoral radiomic features with clinical variables to predict pCR after nICT.

**Materials and Methods:**

We retrospectively analyzed 122 consecutive patients with ESCC who underwent contrast-enhanced computed tomography (CT) before nICT followed by surgical resection. Radiomic features were extracted from manually delineated intratumoral and peritumoral regions, and corresponding radiomic scores were constructed. Independent clinical predictors were combined into a clinical score. These three scores were integrated using multivariable logistic regression to develop a multimodal nomogram. Model performance was evaluated using receiver operating characteristic analysis, calibration curves, and decision curve analysis. Feature contributions were assessed using SHapley Additive exPlanations (SHAP).

**Results:**

Clinical T stage and tumor location constituted the clinical score. The peritumoral radiomic score showed numerically higher AUCs than the intratumoral score in the training (0.818 vs. 0.774) and validation sets (0.808 vs. 0.656). The multimodal nomogram showed good discrimination, with AUCs of 0.901 and 0.862 in the training and validation sets, respectively. SHAP analysis indicated that the peritumoral radiomic score had the largest relative contribution among model components.

**Conclusions:**

A CT-based multimodal nomogram incorporating intratumoral and peritumoral radiomic features with clinical variables showed good performance for preoperative prediction of pCR after nICT in ESCC. Peritumoral radiomic features may provide complementary imaging information, although their biological interpretation remains exploratory.

## Highlights

• A CT-based multimodal nomogram was developed to predict pCR after nICT in ESCC.
• Peritumoral radiomic features may provide complementary imaging information.
• Combining intratumoral, peritumoral, and clinical scores showed good discrimination.

## 1 Introduction

Esophageal squamous cell carcinoma (ESCC) accounts for the majority of esophageal cancer in East Asia, representing more than 90% of cases and ranking among the top four causes of cancer-related death in China [1], [2]. For patients with resectable locally advanced disease, neoadjuvant chemotherapy or, more commonly, neoadjuvant chemoradiotherapy (nCRT) followed by radical esophagectomy constitutes the standard of care, supported by substantial evidence demonstrating improved R0 resection rates, enhanced pathological response, and prolonged survival [3], [4]. A pathological complete response (pCR) has emerged as a robust surrogate endpoint, strongly associated with favorable long-term outcomes [5], [6]. Although nCRT can achieve pCR rates approaching 40% in selected cohorts, its attendant toxicities, strict radiotherapy requirements, and potential delay to surgery limit its widespread applicability [7], [8].

The addition of immune checkpoint inhibitors to cytotoxic chemotherapy, collectively termed neoadjuvant immunochemotherapy (nICT), has recently emerged as an alternative strategy that may offer broader applicability and a more favorable safety profile. Early-phase trials have reported encouraging results; however, the pCR rate with nICT remains around 30%, with marked interpatient heterogeneity [9], [10], [11]. Consequently, a significant proportion of patients are exposed to the toxicity and cost of intensive therapy without achieving maximal benefit, underscoring the need for reliable pretreatment tools to identify likely responders and inform individualized therapeutic strategies.

Although molecular biomarkers such as programmed death-ligand 1 (PD-L1) expression and tumor mutational burden (TMB) have been widely investigated as predictors of immunotherapy response, their clinical utility remains limited [12], [13]. These assays require tissue samples obtained via invasive biopsy and rely on complex, costly molecular analyses, restricting their feasibility in routine practice. Moreover, sampling bias and intratumoral heterogeneity further compromise predictive accuracy, and responses are consistently observed even in patients with negative PD-L1 expression or low TMB [14], [15], [16]. These limitations highlight the need for reliable, broadly accessible, and noninvasive biomarkers to guide patient selection.

In this context, radiomics has emerged as a promising alternative, enabling extraction of high-dimensional, quantitative features from standard-of-care medical imaging in a noninvasive and reproducible manner. Prior studies have demonstrated its utility in staging, treatment response prediction, and survival assessment in ESCC [17], [18], [19], [20]. More recently, computed tomography (CT)-based radiomics models have shown encouraging performance in predicting pCR after nICT [21], [22], [23]. Nonetheless, most existing efforts have focused exclusively on intratumoral features, whereas the peritumoral region, which may provide information on tumor-host interactions and local microenvironmental changes, remains less well explored.

Therefore, we aimed to develop and validate a multimodal predictive model that integrates intratumoral and peritumoral radiomic features from contrast-enhanced CT with relevant clinical variables, in order to noninvasively predict pCR after nICT in patients with locally advanced ESCC.

## 2 Materials and methods

**Fig. 1:**
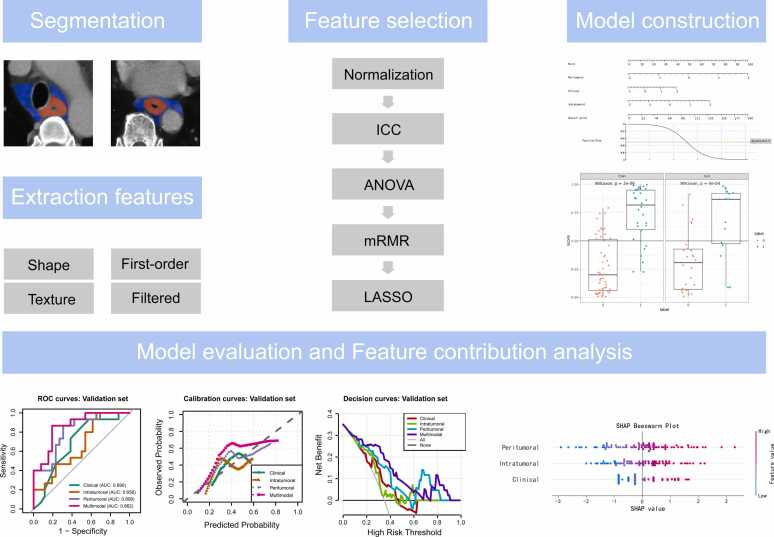
Flowchart illustrating the workflow for model development. ANOVA, analysis of variance; DCA, decision curve analysis; ICC, intraclass correlation coefficient; LASSO, least absolute shrinkage and selection operator; LR, logistic regression; mRMR, minimum redundancy maximum relevance; ROC, receiver operating characteristic curve; SHAP, SHapley Additive exPlanations.

This retrospective study was approved by the Ethics Committee of Peking University Cancer Hospital & Institute, with the requirement for written informed consent waived. The overall workflow of the study is illustrated in Fig. 1.

### 2.1 Patient selection

Eligible patients had pathologically confirmed ESCC and underwent radical esophagectomy following at least one cycle of nICT. Inclusion criteria were as follows: (1) clinical stage T1–3 N + or T3–4aNany (M0/M1 limited to nodal metastases) according to the 8th UICC/AJCC TNM edition; (2) contrast-enhanced chest CT performed within 14 days prior to initiating nICT, with adequate image quality; (3) aged between 18 and 75 years; (4) no history of prior anticancer therapy; and (5) availability of complete postoperative pathological data.

Patients were excluded if they met any of the following criteria: (1) cervical ESCC; (2) multiple primary esophageal cancers or other malignancies; (3) synchronous primary tumors; (4) incomplete (R1 or R2) surgical resection; or (5) poor image quality due to motion artifacts or insufficient tumor coverage.

All patients received nICT consisting of paclitaxel and carboplatin combined with a PD−1/PD-L1 inhibitor (nivolumab, camrelizumab, tislelizumab, toripalimab, sintilimab, durvalumab, or pembrolizumab). The distribution of PD−1/PD-L1 inhibitors is summarized in Supplementary Table 1. Treatment was administered every three weeks for 1–4 cycles prior to surgery. Radical esophagectomy was performed 4–8 weeks after completion of nICT. Resection specimens were evaluated by a dedicated gastrointestinal pathologist and reviewed by a senior pathologist with expertise in esophageal neoplasms. Tumor regression grade (TRG) was assigned according to the College of American Pathologists (CAP) protocol [24]: TRG 0, no residual viable tumor cells; TRG 1, rare residual tumor cells; TRG 2, partial response with residual tumor; and TRG 3, minimal or no tumor regression. pCR was defined as TRG 0, while TRG 1–3 constituted the non-pCR group. This binary outcome served as the outcome variable for model development.

### 2.2 Acquisition of contrast-enhanced CT images

All contrast-enhanced chest CT scans were performed using either a 64-slice multidetector CT (MDCT) scanner (Lightspeed VCT or Discovery 750HD, GE Healthcare, Milwaukee, USA) or a 256-slice MDCT scanner (Brilliance iCT, Philips Healthcare, Cleveland, USA). Acquisition parameters were as follows: tube voltage, 120–140 kVp; tube current, 200–400 mA; rotation time, 0.6 s; detector collimation, 64 × 0.625 mm; and matrix size, 512 × 512. Scans were obtained in the craniocaudal direction from the neck to the renal hilum, starting 55 s after intravenous injection of non-ionic contrast medium (1.5 mL/kg body weight; Omnipaque 300, GE Healthcare) at a rate of 3 mL/s through an antecubital vein using a high-pressure injector. This timing corresponded to the portal venous phase and was used to improve visualization of the tumor and surrounding vascular structures. All patients were scanned using this phase, and the portal venous phase images were used for delineation of both intratumoral and peritumoral ROIs. Sagittal and coronal reconstructions were generated from axial images with slice thickness of 0.625 mm.

### 2.3 Image segmentation

To ensure consistency in spatial resolution, all CT images were resampled to an isotropic voxel size of 1 × 1 × 1 mm³ . Manual segmentation of the regions of interest (ROIs) was performed using 3D Slicer (version 4.10.0; https://download.slicer.org) by two experienced radiologists who were blinded to all clinical and pathological data. ROIs included both the primary tumor and the peritumoral region.

The primary tumor ROI was delineated on the axial slice with the largest tumor cross-sectional area and on two adjacent slices above and below this level. Tumors were identified as focal esophageal wall thickening ≥ 5 mm with contrast enhancement. Contours were drawn slice-by-slice, excluding the esophageal lumen, adjacent vessels, periesophageal fat, and artifacts. A threshold filter was applied to exclude voxels with attenuation < −50 or > 200 Hounsfield units (HU).

The peritumoral ROI was defined as the periesophageal soft tissue surrounding the primary tumor, which includes adjacent adipose tissue, small vessels, lymphatic structures, and lymph nodes. For slices above the level of the carina, the anterior boundary was defined by the anterior wall of the trachea. The lateral boundary extended to the mediastinal pleura or adjacent mediastinal structures, such as the aortic arch or azygos vein. The posterior boundary corresponded to the anterior surface of the vertebral body or prevertebral fascia. For slices below the level of the carina, the anterior boundary was defined by the region between the esophageal wall and the descending aorta. The lateral and posterolateral boundaries extended to the pleural reflection, outer margin of the descending aorta, or the paravertebral groove. Lymph nodes located within the peritumoral region were not intentionally excluded; however, they were not selectively included based on size, morphology, or suspected metastatic status. Gas-containing structures, vertebrae, and large vessels were excluded from the ROI.

Representative examples of intratumoral and peritumoral ROI delineation for tumors located above and below the carina are shown in Supplementary Fig. 1.

### 2.4 Radiomic feature extraction and score construction

Radiomic features were extracted from each ROI using PyRadiomics in accordance with the Image Biomarker Standardization Initiative (IBSI) guidelines [25]. A total of 851 features were extracted per ROI. To reduce variability associated with different CT scanners, ComBat harmonization was applied [26].

All features were normalized to a range between 0 and 1 using the min–max scaling method. To eliminate redundant features and reduce the risk of overfitting, a four-step feature selection strategy was implemented. First, radiomic features with intraclass correlation coefficients (ICCs) greater than 0.8 were retained to ensure robustness and reproducibility [27]. Second, two-sided Student’s *t*-test or Mann–Whitney *U* test was performed to identify features significantly associated with pCR. Third, the minimum redundancy maximum relevance (mRMR) algorithm was applied to prioritize features with maximum relevance and minimum redundancy. Finally, least absolute shrinkage and selection operator (LASSO) regression with 10-fold cross-validation was used to select the optimal subset of predictive features.

Radiomic scores, designated as the intratumoral score and the peritumoral score, were subsequently calculated as linear combinations of the selected features weighted by their corresponding LASSO coefficients.

### 2.5 Clinical score construction

Clinical variables assessed included age, sex, body mass index (BMI), smoking and drinking history, tumor differentiation, tumor location, tumor length, number of treatment cycles, clinical T stage, clinical N stage, and overall clinical TNM stage. Clinical T stage was determined primarily based on pretreatment contrast-enhanced CT imaging. Variables with *P* ≤ 0.05 in univariable logistic regression were subsequently entered into a multivariable logistic regression model to generate the clinical score.

### 2.6 Nomogram construction and evaluation

A multivariable logistic regression model integrating the intratumoral, peritumoral, and clinical scores was developed, with variable selection optimized using the Akaike Information Criterion (AIC). A predictive nomogram was constructed based on this model. To facilitate model interpretability, SHapley Additive exPlanations (SHAP) [28] were used to quantify the contribution of each score to the predicted probability of pCR. SHAP values were ranked to describe the relative influence of the model components.

### 2.7 Statistical analysis

A sample size of 15 patients in the pCR group and 23 in the non-pCR group was determined to provide 81% power to detect a difference of 0.25 between an area under the receiver operating characteristic curve (AUC) of 0.60 under the null hypothesis and an AUC of 0.85 under the alternative hypothesis, using a two-sided z test at a significance level of 0.05. All statistical analyses were performed using SPSS software (version 26.0; IBM Corp., Armonk, NY, USA) and R software (version 4.3.1; R Foundation for Statistical Computing, Vienna, Austria). Continuous variables were compared using the Student’s *t*-test or Mann–Whitney *U* test, as appropriate, while categorical variables were compared using the chi-square test or Fisher’s exact test. Receiver operating characteristic (ROC) analysis was used to calculate the AUC, and AUCs were compared using DeLong’s test. Sensitivity, specificity, and accuracy were calculated according to optimal cutoff values determined by the Youden index. Internal validation of the combined nomogram was performed using 1000 bootstrap resamples. Calibration was assessed using calibration plots, and clinical utility was evaluated by decision curve analysis (DCA) [29]. A two-sided *P* < 0.05 was considered statistically significant.

## 3 Results

### 3.1 Baseline characteristics

A total of 122 patients were included and randomly assigned to the training (n = 81) and validation (n = 41) sets at a 2:1 ratio. The baseline clinical and pathological characteristics of the two cohorts are summarized in Table 1. The majority were male (n = 102, 83.6%), and most were diagnosed with stage III disease (n = 85, 69.7%). The overall pCR rate was 37.0% (30/81) in the training set and 36.6% (15/41) in the validation set. No significant differences were observed between the pCR and non-pCR groups in either cohort, except for tumor location and clinical T stage, which were significantly different in both sets. The distribution of CT scanner types between the training and validation sets is summarized in Supplementary Table 2.

**Table 1:** Baseline characteristics of patients in training and validation sets.

**Variables**	**Training set (n = 81)**		***P*****value**	**Validation set (n = 41)**		***P*****value**
	**non-pCR**	**pCR**		**non-pCR**	**pCR**	
Sex (%)			0.424			0.231
Male	44 (86.3)	23 (76.7)		24 (92.3)	11 (73.3)	
Female	7 (13.7)	7 (23.3)		2 (7.7)	4 (26.7)	
Age, mean (SD), y	61 ± 7	63 ± 7	0.168	61 ± 6	61 ± 6	0.977
Smoke status (%)			1.000			0.871
Never	17 (33.3)	10 (33.3)		5 (19.2)	4 (26.7)	
Former or Current	34 (66.7)	20 (66.7)		21 (80.8)	11 (73.3)	
Drinking status (%)			0.713			0.399
Never	22 (43.1)	15 (50.0)		9 (34.6)	8 (53.3)	
Former or Current	29 (56.9)	15 (50.0)		17 (65.4)	7 (46.7)	
BMI, median (IQR), kg/m²	23.2 (22.2, 25.8)	23.8 (21.4, 26.3)	0.922	22.8 (21.4, 25.9)	23.2 (19.9, 24.3)	0.839
Tumor location (%)			**0.043**			**0.031**
Upper	3 (5.9)	7 (23.3)		1 (3.8)	4 (26.7)	
Middle	23 (45.1)	14 (46.7)		11 (42.3)	8 (53.3)	
Lower	25 (49.0)	9 (30.0)		14 (53.8)	3 (20.0)	
Tumor differentiation (%)			0.876			0.905
Well	5 (9.8)	4 (13.3)		3 (11.5)	2 (13.3)	
Middle	31 (60.8)	18 (60.0)		19 (73.1)	10 (66.7)	
Poor	15 (29.4)	8 (26.7)		4 (15.4)	3 (20.0)	
Tumor length, mean (SD), mm	48.8 ± 16.0	50.9 ± 20.7	0.620	46.2 ± 14.8	53.6 ± 21.8	0.205
cT^a^ (%)			**0.034**			**0.035**
2	3 (5.9)	7 (23.3)		2 (7.7)	6 (40.0)	
3	48 (94.1)	23 (76.7)		24 (92.3)	9 (60.0)	
cN^a^ (%)			0.209			0.072
0	4 (7.8)	4 (13.3)		0 (0.0)	2 (13.3)	
1	12 (23.5)	12 (40.0)		13 (50.0)	3 (20.0)	
2	33 (64.7)	14 (46.7)		12 (46.2)	10 (66.7)	
3	2 (3.9)	0 (0.0)		1 (3.8)	0 (0.0)	
cTNM^a^ stage (%)			0.370			0.411
2	5 (9.8)	4 (13.3)		1 (3.8)	2 (13.3)	
3	43 (84.3)	26 (86.7)		24 (92.3)	13 (86.7)	
4	3 (5.9)	0 (0.0)		1 (3.8)	0 (0.0)	
NICT cycle (%)			0.597			0.748
1	1 (2.0)	1 (3.3)		1 (3.8)	0 (0.0)	
2	39 (76.5)	26 (86.7)		21 (80.8)	13 (86.7)	
3	8 (15.7)	2 (6.7)		3 (11.5)	2 (13.3)	
4	3 (5.9)	1 (3.3)		1 (3.8)	0 (0.0)	

Abbreviation: BMI, body mass index; IQR, interquartile range; NICT, neoadjuvant immunochemotherapy; pCR, pathological complete response; SD, standard deviation.

^a^Cancer staging was done with the American Joint Committee on Cancer TNM staging system (8th edition).

### 3.2 Clinical score

Univariable and multivariable logistic regression identified clinical T stage and tumor location as independent predictors of pCR (Table 2). These two variables were used to construct the clinical score, which yielded an AUC of 0.702 (95% confidence interval [CI], 0.587–0.817) in the training set and 0.689 (95% CI, 0.525–0.855) in the validation set.

**Table 2:** Univariable and multivariable logistic regression analysis for prediction of pCR.

**Characteristics**	**Univariable analysis**	***P*****value**	**Multivariable analysis**	***P*****value**
	**OR (95%CI)**		**OR (95%CI)**	
Clinical score	1.602 (1.266–2.027)	< 0.001	1.412 (1.020–1.783)	0.047
Intratumoral score	3.173 (1.755–5.738)	< 0.001	3.084 (1.463–6.502)	0.003
Peritumoral score	3.513 (1.897–6.503)	< 0.001	3.384 (1.691–6.770)	< 0.001

Abbreviations: CI, confidence interval; OR, odds ratio; pCR, pathological complete response.

### 3.3 Intratumoral and peritumoral radiomic scores

Feature selection was performed separately for radiomic features extracted from the intratumoral and peritumoral ROIs. After dimensionality reduction, two intratumoral and five peritumoral radiomic features associated with pCR were retained. The radiomic score for each patient was calculated as a linear combination of the selected features weighted by their corresponding coefficients. The formulas were defined as follows:

Intratumoral score = 0.536294 × tumor-original_glcm_DifferenceAverage + 0.463706 × tumor-wavelet-HLL_ngtdm_Contrast

Peritumoral score = 0.182284 × peritumor-wavelet-HHH_gldm_DependenceVariance + 0.301197 × peritumor-wavelet-HHH_gldm_SmallDependenceHighGrayLevelEmphasis + 0.194417 × peritumor-wavelet-HHH_glcm_ClusterTendency + 0.160796 × peritumor-wavelet-LHL_glrlm_LongRunLowGrayLevelEmphasis + 0.161305 × peritumor-wavelet-HHL_glcm_Idm

The peritumoral score showed numerically higher AUCs than the intratumoral score in both the training set (0.818 [95% CI, 0.723–0.913] vs. 0.774 [95% CI, 0.670–0.878]) and the validation set (0.808 [95% CI, 0.672–0.944] vs. 0.656 [95% CI, 0.485–0.828]), although these differences were not statistically significant (all *P* > 0.05).

### 3.4 Nomogram construction and performance evaluation

**Fig. 2:**
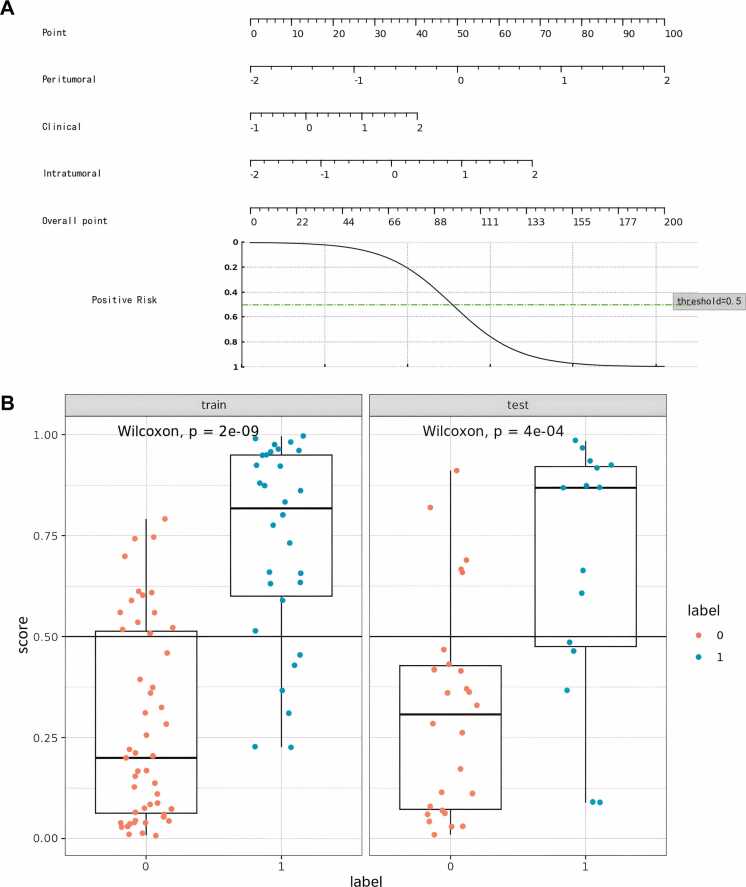
Predictive value of the multimodal nomogram. **(A)** Nomogram integrating clinical, intratumoral, and peritumoral scores for predicting pCR. **(B)** Distribution of nomogram-predicted scores in the training and validation sets. pCR, pathological complete response.

**Fig. 3:**
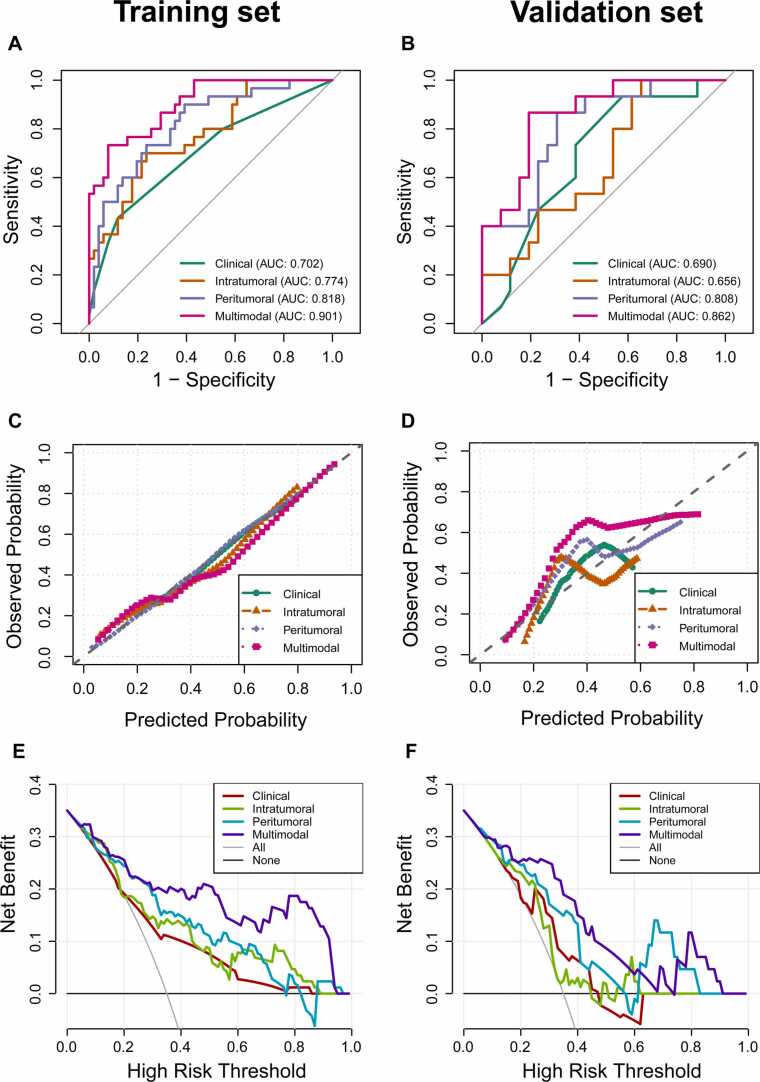
Performance of the predictive models in the training and validation sets. **(A, B)** ROC curves for each model in the training (**A**) and validation (**B**) sets. **(C, D)** Calibration curves for each model in the training (**C**) and validation (**D**) sets. **(E, F)** DCA illustrating the net clinical benefit of each model in the training (E) and validation (**F**) sets. ROC, receiver operating characteristic; DCA, decision curve analysis.

A multivariable logistic regression model integrating the clinical, intratumoral, and peritumoral scores was developed to generate the final predictive nomogram for pCR (Fig. 2). Internal validation using bootstrap resampling yielded an AUC of 0.900 (95% CI, 0.839–0.948) in the training set. The multimodal model showed higher discrimination than the individual models, with an AUC of 0.901 (95% CI, 0.836–0.966) in the training set and 0.862 (95% CI, 0.749–0.974) in the validation set (Fig. 3**A**–**B**). Pairwise comparisons using DeLong’s test confirmed statistically significant improvements over individual models, except when compared with the peritumoral score alone in the validation set (*P* > 0.05). Calibration curves demonstrated good agreement between predicted and observed probabilities in both the training and validation sets (Fig. 3**C**–**D**). Decision curve analysis further indicated that the multimodal model provided a higher net clinical benefit across a range of threshold probabilities compared with the individual models (Fig. 3**E**–**F**).

The multimodal model demonstrated good diagnostic performance. In the training set, it achieved an accuracy of 85.2%, sensitivity of 73.3%, specificity of 92.2%, positive predictive value (PPV) of 84.6%, and negative predictive value (NPV) of 85.5%. In the validation set, accuracy was 73.2%, sensitivity 53.3%, specificity 84.6%, PPV 66.7%, and NPV 75.9%, indicating that the model maintained relatively high specificity, although sensitivity was lower than in the training set.

### 3.5 Feature contribution analysis

**Fig. 4:**
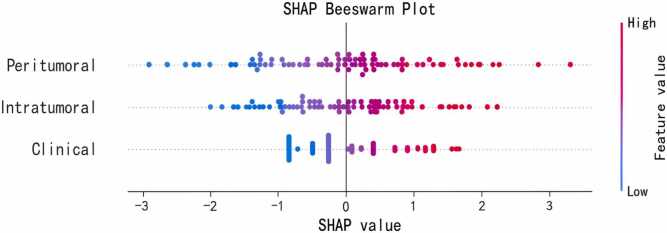
SHAP summary plot of the multimodal model. Each dot represents the contribution of an individual feature to the predicted probability of pCR for a single patient. The x-axis indicates SHAP values, reflecting the direction and magnitude of each feature’s contribution to the model output. Features are ranked by their relative importance on the y-axis. Red denotes higher feature values associated with increased likelihood of pCR; blue denotes lower values associated with reduced likelihood. pCR, pathological complete response; SHAP, SHapley Additive exPlanations.

SHAP analysis was performed to assess the relative contribution of each component score. As illustrated in Fig. 4, the peritumoral score exhibited the widest SHAP value distribution, indicating the largest relative contribution to the model output. Higher SHAP values (red) were associated with an increased probability of achieving pCR, whereas lower values (blue) indicated a reduced likelihood of response.

## 4 Discussion

Accurate preoperative prediction of pathological response plays a critical role in optimizing treatment decisions for patients with locally advanced ESCC receiving nICT. In this study, we developed and validated a multimodal predictive model that incorporates pretreatment radiomic features from both intratumoral and peritumoral regions, along with key clinical variables, to noninvasively predict pCR. The model showed good discriminative performance in both the training and validation sets. SHAP analysis indicated that the peritumoral radiomic score had the largest relative contribution among the model components.

Our results showed that radiomic features derived from the peritumoral region demonstrated numerically higher predictive performance for pCR after nICT than intratumoral features, although the difference did not reach statistical significance. These findings suggest that the peritumoral microenvironment may contain additional imaging information related to treatment response. The peritumoral region represents the interface between the tumor and surrounding host tissues, where vascular, lymphatic, and stromal components interact and may influence tumor progression and therapeutic sensitivity. Previous studies have suggested that radiomic features extracted from peritumoral tissues may be associated with biological processes related to the tumor microenvironment, including immune activity and stromal remodeling, which may not be fully captured by tumor-intrinsic imaging characteristics alone [30], [31]. In addition, the surrounding periesophageal soft tissue may contain adipose components that influence drug metabolism and treatment response through paracrine signaling and extracellular matrix remodeling [32]. Such microenvironmental alterations may generate subtle spatial heterogeneity in CT images that is not readily appreciable on visual assessment but can be quantified using radiomic analysis [33]. Consequently, peritumoral radiomic features may partly reflect tumor–host interactions and treatment-related biological responses. These findings should be interpreted as imaging-level associations rather than evidence that the peritumoral signature directly represents a specific biological process. From a clinical perspective, incorporating peritumoral radiomic information may provide complementary insights beyond tumor-centered imaging and may be useful for preoperative risk stratification in patients undergoing nICT.

Our findings are consistent with emerging radiomics studies investigating the role of peritumoral features in predicting treatment response in ESCC. Previous studies by Hu et al. and Li et al. demonstrated that incorporating peritumoral radiomic features significantly improved the prediction of pCR following neoadjuvant chemoradiotherapy [30], [34]. However, the definition of the peritumoral region varies across studies. Hu et al. manually delineated the peritumoral region to include adjacent periesophageal soft tissue and lymph nodes while excluding surrounding structures such as the airway, aorta, vertebrae, and azygos vein. In contrast, Li et al. defined the peritumoral region using a fixed morphological expansion of 6 mm from the tumor boundary. In the present study, we adopted a delineation strategy similar to that described by Hu et al. Because the esophagus is located within the mediastinum and surrounded by multiple anatomical structures, simple geometric expansion may inadvertently include tissues unrelated to tumor biology, potentially introducing noise into radiomic feature extraction. Therefore, we defined the peritumoral region according to anatomical boundaries to better characterize the surrounding periesophageal soft tissue. In addition, segmentation was performed on the maximal axial slice ± 2 slices rather than the entire tumor volume to balance segmentation efficiency and clinical feasibility. Previous work by Shi et al. suggested that two-dimensional radiomics models may achieve comparable or even superior performance to three-dimensional models in predicting pCR in ESCC patients undergoing neoadjuvant immunochemoradiotherapy [35].

Currently, treatment response is primarily assessed through modalities such as endoscopy or CT-based response evaluation [36], [37], which are only applicable after neoadjuvant treatment has commenced. This limits their utility in guiding initial therapeutic decisions. Our findings support the role of baseline clinical T stage and tumor location as useful predictors of pCR, consistent with prior studies [38], [39]. Specifically, patients with clinical T2 tumors were more likely to achieve pCR than those with T3 disease. Similarly, tumors located in the upper or middle thoracic esophagus were associated with higher pCR rates than lower esophageal lesions. This may reflect anatomic differences in perfusion, with the upper esophagus receiving more robust arterial blood supply, potentially enhancing drug delivery and response [39].

Despite the promising results, several limitations should be acknowledged. First, this was a retrospective, single-center study with a relatively limited sample size, particularly in the validation cohort (n = 41), which may limit the generalizability of our findings and increase the risk of model optimism. Although an independent validation cohort and bootstrap-based internal validation were included, further prospective validation in larger, multicenter cohorts is still needed. In addition, treatment heterogeneity may also have influenced the results, as different PD−1/PD-L1 inhibitors were used and the number of neoadjuvant treatment cycles varied across the cohort. Although this reflects routine clinical practice, such variation may have affected pathological response and introduced additional variability into model performance. While the model demonstrated good discrimination, the sensitivity observed in the validation cohort was moderate, indicating that some pCR cases may not be fully captured by the model. Therefore, the proposed model should be interpreted as a complementary tool for preoperative risk stratification rather than a standalone decision-making approach. Second, tumor segmentation was performed on the maximal axial slice ± 2 slices rather than the whole-tumor volume. While this 2D strategy improves efficiency and may enhance interobserver consistency, it may not fully capture cranio-caudal tumor heterogeneity. Future studies incorporating automated 3D whole-tumor segmentation may provide more comprehensive spatial characterization. Third, the biological underpinnings of radiomic features remain incompletely understood. Further studies are needed to clarify their radiogenomic and immunological relevance. Lastly, integrating radiomic features with molecular and genomic biomarkers may further improve predictive performance and support more individualized treatment planning in ESCC.

## 5 Conclusion

Peritumoral radiomic features may provide complementary information beyond intratumoral features for predicting response to nICT in patients with ESCC. The proposed multimodal model, integrating intratumoral and peritumoral radiomic features with relevant clinical variables, showed good performance for preoperative prediction of pCR. This noninvasive, image-based approach may support preoperative risk stratification, although further multicenter validation and biological correlation studies are needed before clinical application.

## Ethics

This retrospective study was approved by the the Ethics Committee of Peking University Cancer Hospital & Institute. The requirement for written informed consent was waived due to the retrospective nature of the study.

## CRediT authorship contribution statement

**Haitao Zhu:** Writing – original draft, Validation, Methodology, Data curation, Conceptualization. **Yaqi Wang:** Writing – original draft, Visualization, Validation, Project administration, Formal analysis. **Xiaoting Li:** Writing – review & editing, Data curation. **Xinrun Cui:** Writing – review & editing, Data curation. **Bo Zhao:** Writing – original draft, Software, Methodology, Formal analysis, Conceptualization. **Nan Wu:** Writing – review & editing, Supervision, Resources. **Shaolei Li:** Writing – review & editing. **Yingshi Sun:** Writing – review & editing, Supervision, Funding acquisition.

## Funding

This work was supported by grants from the Beijing Hospitals Authority Clinical Medicine Development of Special Funding Support (ZLRK202522), 10.13039/501100004826Beijing Natural Science Foundation (7244511), the Science Foundation of Peking University Cancer Hospital (XKFZ2613 and ZY202508), and the Noncommunicable Chronic Diseases–National Science and Technology Major Project (2023ZD0512400).

## Declaration of Competing Interest

The authors declare that they have no known competing financial interests or personal relationships that could have appeared to influence the work reported in this paper.

## Appendix A Supplementary material

Supplementary material

Supplementary material.Fig.lementary Fig. 1. Representative examples of intratumoral and peritumoral ROI delineation on contrast-enhanced CT images. (**A**) Representative case of a tumor located above the level of the carina, showing the intratumoral ROI in red and the peritumoral ROI in blue. (B) Representative case of a tumor located below the level of the carina, showing the intratumoral ROI in red and the peritumoral ROI in blue. CT, computed tomography; ROI, region of interest
