# Supplementary material for: Intratumoral and peritumoral radiomics for preoperative prediction of pathological complete response to neoadjuvant immunochemotherapy in patients with esophageal squamous cell carcinoma
Source: Eur J Radiol Open. 2026 Jun 17;17:100774. doi: 10.1016/j.ejro.2026.100774 (PMC13311210; doi:10.1016/j.ejro.2026.100774)
Supplement: Supplementary file 1 — Supplementary material [file mmc1.docx]

**Supplementary Table 1.** Distribution of PD-1/PD-L1 inhibitors in the training and validation sets^*^.

| **Variables** | **Training set (n = 81)** | | ***P* value** | **Validation set (n=41)** | | ***P* value** |
| --- | --- | --- | --- | --- | --- | --- |
|  | **non-pCR** | **pCR** |  | **non-pCR** | **pCR** |  |
| PD-1/PD-L1 inhibitors |  |  | 0.548 |  |  | 0.485 |
| Pembrolizumab | 2 (3.9) | 1 (3.3) |  | 1 (3.8) | 1 (6.7) |  |
| Camrelizumab | 2 (3.9) | 2 (6.7) |  | 2 (7.7) | 0 (0.0) |  |
| Toripalimab | 11 (21.6) | 7 (23.3) |  | 9 (34.6) | 2 (13.3) |  |
| Tislelizumab | 20 (39.2) | 12 (40.0) |  | 7 (26.9) | 6 (40.0) |  |
| Sintilimab | 9 (17.6) | 3 (10.0) |  | 5 (19.2) | 3 (20.0) |  |
| Nivolumab | 7 (13.7) | 3 (10.0) |  | 2 (7.7) | 2 (13.3) |  |
| Durvalumab | 0 (0.0) | 2 (6.7) |  | 0 (0.0) | 1 (6.7) |  |

^*^ All patients received neoadjuvant immunochemotherapy consisting of paclitaxel and carboplatin combined with one PD-1/PD-L1 inhibitor.

Abbreviation: PD-1, programmed cell death protein 1; PD-L1, programmed death-ligand 1.

**Supplementary Table 2.** Distribution of CT scanners in the training and validation sets^*^.

| **Scanner type** | **Manufacturer** | **Training set (n = 81)** | **Validation set (n = 41)** |
| --- | --- | --- | --- |
| 64-slice MDCT (Lightspeed VCT) | GE Healthcare | 30 | 15 |
| 64-slice MDCT (Discovery 750HD) | GE Healthcare | 20 | 10 |
| 256-slice MDCT (Brilliance iCT) | Philips Healthcare | 31 | 16 |

^*^ All scans were performed with contrast-enhanced chest CT.

Abbreviation: MDCT, multidetector computed tomography.
