# Supplementary figures and images for: Intratumoral and peritumoral radiomics for preoperative prediction of pathological complete response to neoadjuvant immunochemotherapy in patients with esophageal squamous cell carcinoma
Source: Eur J Radiol Open. 2026 Jun 17;17:100774. doi: 10.1016/j.ejro.2026.100774 (PMC13311210; doi:10.1016/j.ejro.2026.100774)

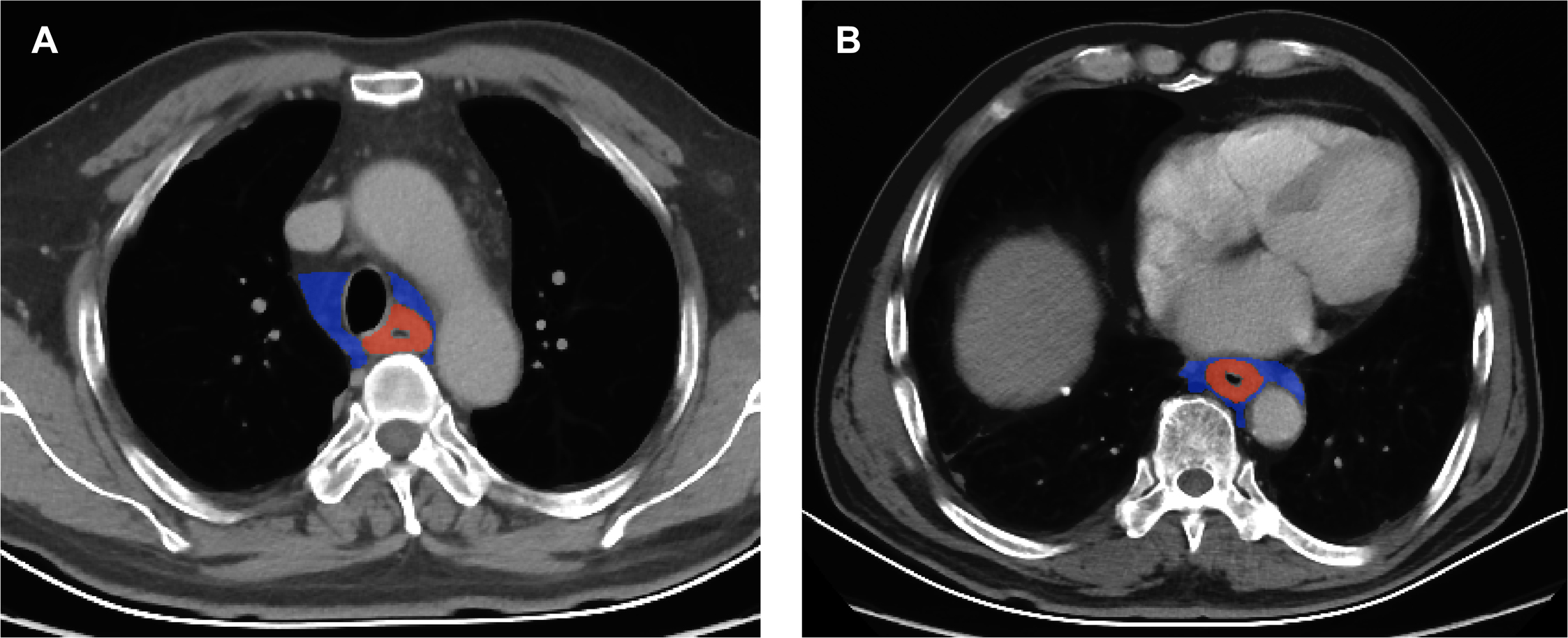

Supplement: Supplementary file 2 — Supplementary material.Fig.lementary Fig. 1. Representative examples of intratumoral and peritumoral ROI delineation on contrast-enhanced CT images. (A) Representative case of a tumor located above the level of the carina, showing the intratumoral ROI in red and the peritumoral ROI in blue. (B) Representative case of a tumor located below the level of the carina, showing the intratumoral ROI in red and the peritumoral ROI in blue. CT, computed tomography; ROI, region of interest [file mmc2.jpg]
